# Flare of bullous lupus erythematosus in patients treated with anifrolumab for discoid lupus erythematosus: 2 cases

**DOI:** 10.1016/j.jdcr.2024.11.045

**Published:** 2025-02-12

**Authors:** Cécile Lesort, Anne-Laure Breton, Chloé Wirbel, Cécile-Audrey Durel, Marine Chastagner, Emmanuel Ribereau-Gayon, Arnaud Hot, Jean Kanitakis

**Affiliations:** a Department of Dermatology, Hospices Civils de Lyon, Edouard Herriot Hospital, Lyon, France; b Department of Dermatology, Saint Luc Saint Joseph Hospital, Lyon, France; c Department of Internal Medicine, Saint Luc Saint Joseph Hospital, Lyon, France; d Department of Internal Medicine, Hospices Civils de Lyon, Lyon, France; e Department of Pathology, Hospices Civils de Lyon, Lyon Sud Hospital Center, Pierre Bénite, France

**Keywords:** anifrolumab, biologics, bullous systemic lupus erythematosus, cutaneous lupus erythematosus, induced lupus

## Introduction

Anifrolumab is a novel IFNAR1 antagonist recently approved for the treatment of moderate-to-severe systemic lupus erythematosus (SLE), especially for patients with severe and/or refractory cutaneous manifestations.^1^^,^^2^ We report here 2 patients with SLE who developed after the first dose of anifrolumab, a bullous eruption histologically diagnosed as bullous lupus erythematosus (LE). None of these patients had prior bullous manifestations.

## Case reports

**Fig 1:**
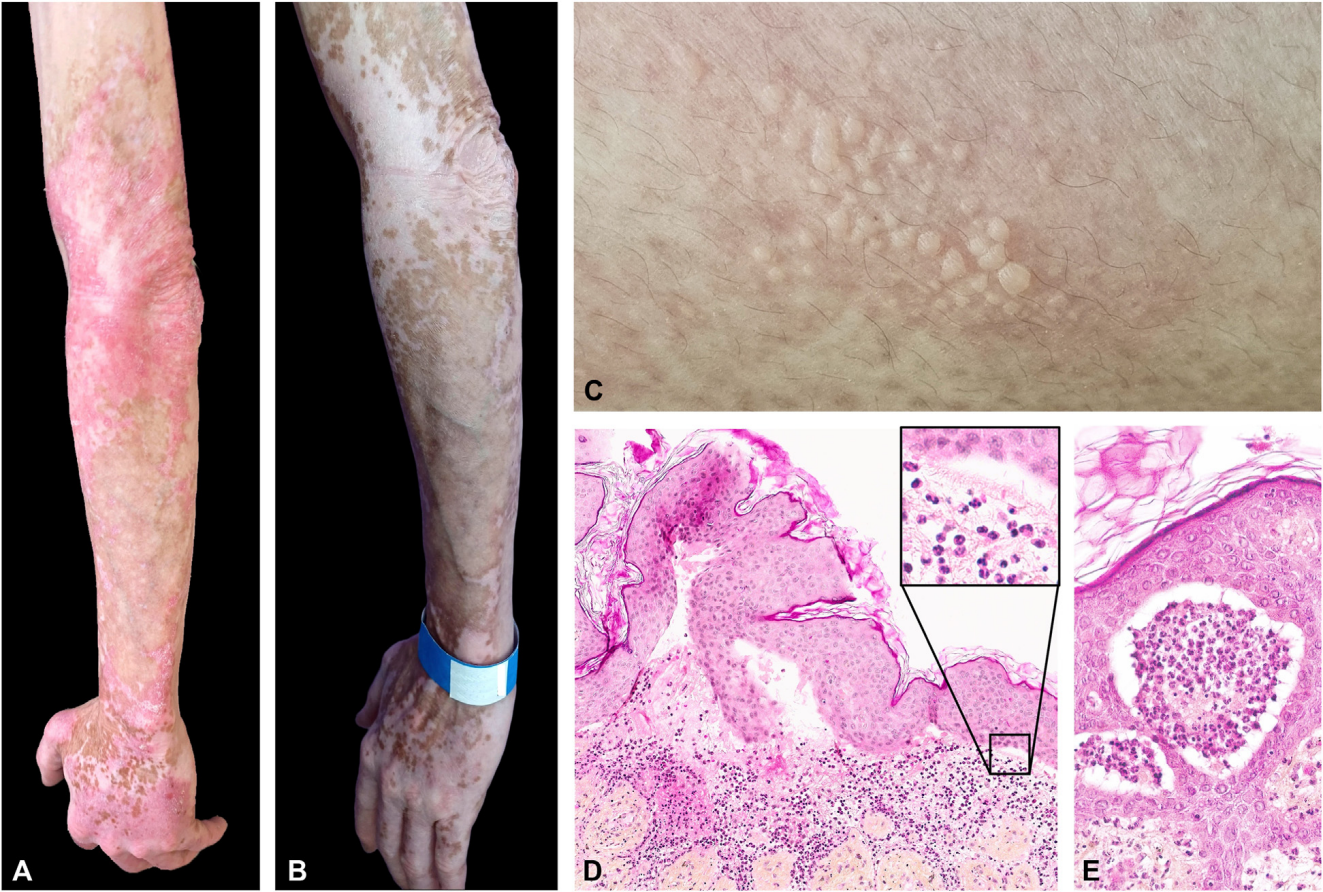
Patient 1: Discoid cutaneous lupus before (**A**) and 4 months after treatment with anifrolumab (**B**); bullous lupus erythematosus a few days after first infusion of anifrolumab (**C**); histological examination (hematoxylin-eosin-saffron stain) (**D**) showing a dense perivascular, predominantly neutrophilic infiltrate in the dermis with subepidermal blisters containing neutrophils (**E**).

Patient 1 is a 40-year-old man with SLE since 2007. He had been initially treated for class IV nephropathy with corticosteroids and cyclophosphamide for 2 years with no recurrence. He was treated in our department for cutaneous and articular lupus manifestations. He presented with a severe, highly-photosensitive rash of the face, years, arms, and trunk. He had erythematous, violaceous, scaly lesions with atrophic and dyschromic scars and was diagnosed with chronic/discoid cutaneous LE (histologically confirmed). Despite satisfactory control of his joint manifestations, his skin lesions remained refractory to multiple therapeutic lines (hydroxychloroquine, methotrexate, mycophenolate mofetil, and azathioprine). He had persistent, active cutaneous lesions despite a combined treatment with hydroxychloroquine, thalidomide, and belimumab (CLASI [Cutaneous LE Disease Areas and Activity Index]-A score 24) (Fig 1, *A*). Thalidomide and belimumab were discontinued, and anifrolumab was initiated.

Ten days after the first anifrolumab infusion, he developed erythematous and bullous skin lesions on the trunk (Fig 1, *C*). Histological examination of a skin biopsy (Fig 1, *D*) showed neutrophilic microabscesses in the tips of dermal papillae and subepidermal blisters containing neutrophils (Fig 1, *E*). A dense perivascular, predominantly neutrophilic infiltrate was seen in the mid-dermis. Alcian blue staining showed increased amounts of dermal mucin. Direct immunofluorescence examination showed granular, discontinuous deposits of IgG, IgA, and C3 at the dermal-epidermal junction. Polymerase chain reaction for herpes simplex virus and varicella zona virus was negative. These findings were diagnostic of bullous cutaneous LE. Autoantibodies to type VII collagen were not detected. Remarkably, spectacular improvement of the discoid lesions was achieved (CLASI [Cutaneous LE Disease Area and Activity Index]-A score 6). Given the improvement of discoid lesions (Fig 1, *B*), anifrolumab was continued. The bullous lesions resolved with high topical corticosteroids. No recurrence developed after 4 subsequent infusions.

**Fig 2:**
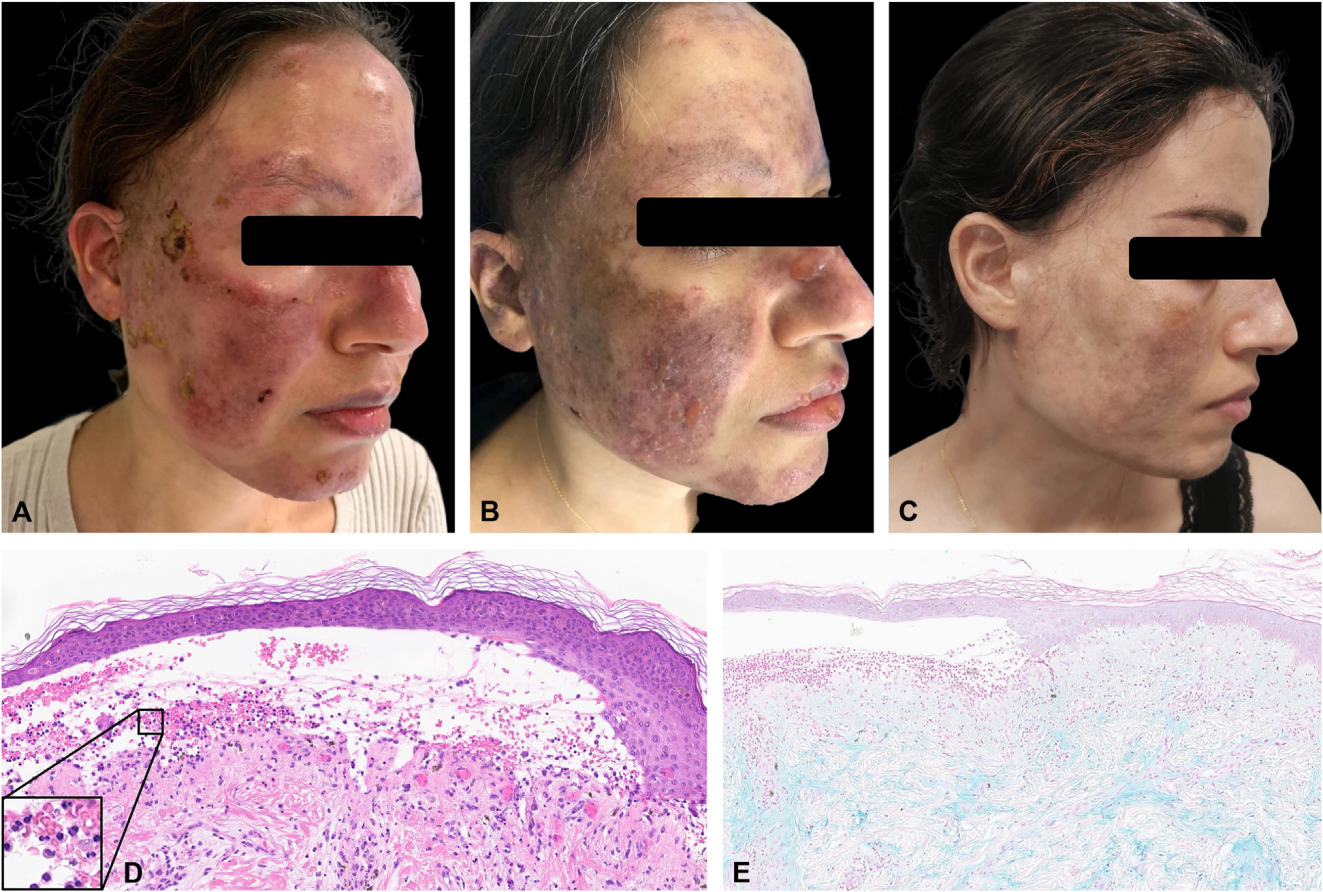
Patient 2: Discoid cutaneous lupus before treatment (**A**); bullous lupus erythematosus a few days after first infusion of anifrolumab (**B**); 6 months after treatment with anifrolumab (**C**); histological examination (hematoxylin-eosin-saffron stain) (**D**) showing subepidermal blisters containing neutrophils with neutrophils present diffusely within papillary and superficial reticular dermis and mucin deposits stained with Alcian blue (**E**).

Patient 2 is a 37-year-old woman with a 10-year history of systemic cutaneous and articular LE. She was treated with methotrexate 15 mg/week, prednisone 20 mg/day, and hydroxychloroquine 400 mg/day. Despite this treatment, she had persistent cutaneous involvement with diffuse erythematous, erosive and scaly lesions, along with atrophic scars of the face, ears, and hands (CLASI-A score 26) (Fig 2, *A*). She also had oral mucosa ulcers, areas of scalp nonscarring alopecia, and cortico-dependence of arthralgias. Treatment with belimumab was started but had to be stopped after a single infusion because of gastrointestinal side effects. Treatment with thalidomide was discontinued after 5 days due to drowsiness. Given the patient's progressive disease, treatment with anifrolumab was initiated. Three days after the first infusion of anifrolumab, bullous lesions developed on the face (Fig 2, *B*). Skin biopsy showed a predominantly neutrophilic inflammatory infiltrate of the upper dermis, dermal edema, and subepidermal blisters containing neutrophils (Fig 2, *D*) with mucin deposits stained with Alcian blue (Fig 2, *E*). Autoantibodies to type VII collagen were not detected. Similar to patient 1, cutaneous and mucosal manifestations improved (Fig 2, *C*). In addition to her dermatological bullous condition, the patient continued to present disabling inflammatory arthralgia, which led us to temporarily increase the oral corticosteroid therapy dosage. Topical corticosteroids were also added, resulting in resolution of the flare-up, without the need of dapsone administration. The patient had a few small bullous lesions over 2 months with no recurrence since, with no other skin and rheumatologic LE activity. The systemic corticosteroid therapy was rapidly tapered to 5 mg/d.

## Discussion

A few cases of induced bullous LE have been reported in the literature in patients otherwise treated for systemic LE, for example, with hydralazine^3^ (known to trigger drug-induced LE), with no clear hypothesis put forward regarding the pathophysiology of this type of reaction.

Bullous LE is rare.^4^ Although bullous lesions can develop during the course of LE, particularly in acute and subacute cutaneous forms, due to the severity of interface dermatitis,^5^ like in Rowell syndrome, which can even mimic Stevens-Johnson or toxic epidermal necrolysis syndrome,^6^ our patients presented with lesions suggestive of authentic bullous LE with histological evidence of a neutrophilic infiltrate. Neither of them had prior bullous lesions.

Some studies have reported the coinvolvement of interferon and Th17 pathways^7^ in systemic LE and the interaction between neutrophils and Th17 cells.^8^ By synthesizing IL17-A, Th17 cells activate neutrophils, in particular by increasing the synthesis of G colony-stimulating factor and thus the maturation and mobilization of neutrophil polynuclear cells. A physiopathological hypothesis for the triggering of these bullous lesions could be an acute paradoxical neutrophilic activation via the Th17 pathway due to the blockade of the interferon receptor by anifrolumab.

Anifrolumab is a new therapeutic option for severe and refractory cutaneous lesions associated with SLE. Viral infections, especially herpes zoster, are the most commonly reported adverse events.^2^ Here, we report the first observation of bullous flare of cutaneous LE under anifrolumab. In our 2 patients, the lesions resolved without the need for dapsone, the reference treatment for bullous LE, and notably without recurrence during subsequent infusions. While further observations are needed in order to gain further insight into the impact of this reaction, it seems that the occurrence of this type of lesion should not contraindicate the continuation of treatment.

## Conflicts of interest

None disclosed.
